# A Novel Passive Indoor Localization Method by Fusion CSI Amplitude and Phase Information

**DOI:** 10.3390/s19040875

**Published:** 2019-02-20

**Authors:** Xiaochao Dang, Xiong Si, Zhanjun Hao, Yaning Huang

**Affiliations:** 1 College of Computer Science and Engineering, Northwest Normal University, Lanzhou 730070, China; dangxc@nwnu.edu.cn (X.D.); sx2016@nwnu.edu.cn (X.S.); bellanwnu@126.com (Y.H.); 2 Gansu Province Internet of Things Engineering Research Center, Lanzhou 730070, China

**Keywords:** indoor localization, channel state information, device-free passive, WiFi fingerprint, naive Bayes classification, feature fusion

## Abstract

With the rapid development of wireless network technology, wireless passive indoor localization has become an increasingly important technique that is widely used in indoor location-based services. Channel state information (CSI) can provide more detailed and specific subcarrier information, which has gained the attention of researchers and has become an emphasis in indoor localization technology. However, existing research has generally adopted amplitude information for eigenvalue calculations. There are few research studies that have used phase information from CSI signals for localization purposes. To eliminate the signal interference existing in indoor environments, we present a passive human indoor localization method named FapFi, which fuses CSI amplitude and phase information to fully utilize richer signal characteristics to find location. In the offline stage, we filter out redundant values and outliers in the CSI amplitude information and then process the CSI phase information. A fusion method is utilized to store the processed amplitude and phase information as a fingerprint database. The experimental data from two typical laboratory and conference room environments were gathered and analyzed. The extensive experimental results demonstrate that the proposed algorithm is more efficient than other algorithms in data processing and achieves decimeter-level localization accuracy.

## 1. Introduction

Location-based applications and services are concerned with people’s daily lives, and thus have attracted increasing attention. According to different target environments, positioning services can be divided into two situations: outdoor localization and indoor localization. In the outdoor environment, traditional satellite positioning technologies, such as the Chinese BeiDou navigation satellite system (BDS), the global positioning system (GPS), and cellular-based station positioning technology provide highly-precise positioning services that can satisfy the needs of outdoor environment location services [1]. Indoor localization, as its name implies, is the positioning of the target person or object within an indoor environment, such as intrusion detection [2], security monitoring [3], and indoor navigation [4], among others [5]. Indoor localization requires timeliness, accuracy, and stability. However, in an indoor environment, the signal transmission will be limited by multipath interference, the shadow effect, power attenuation, transmission delay, etc., which lead to a poor performance of the positioning service [6]. Based on the above constraints, WiFi [7], Bluetooth [8], Radio Frequency Identification (RFID) [9], ultra-wideband (UWB) [10], and other wireless signal localization methods have been widely researched and applied [11]. 

Unfortunately, Bluetooth, RFID, UWB, and other wireless signal localization methods have their own shortcomings like requiring costly specific devices, being easily affected by external condition factors, or the equipment deployment being too complex. As WiFi technology matures and such devices are popularized, many indoor localization systems based on WiFi signals are being widely used to provide accurate and efficient location services because of the low cost, large range of signal transmission, and strong applicability [12,13,14]. Researchers have obtained received signal strength indicator (RSSI) signals from WiFi devices, analyzed the fluctuations caused by signal changes, established a signal propagation model, and mapped the RSSI signals to a distance, which serve as the basis for indoor localization [15,16]. Although the RSSI-based method has been greatly improved, the disadvantages of the coarse granularity and instability of the RSSI signal restrict the positioning effect [17].

With the use of orthogonal frequency division multiplexing (OFDM) systems and multiple-input multiple-output (MIMO) systems in the 802.11a/n protocol, channel state information (CSI) signals can be extracted from commercial WiFi equipment. In contrast to RSSI signals, which provide only amplitude information, CSI signals can provide both the subcarrier phase and amplitude information as well as better descriptions of the signal changes from the transmitter to the receiver than those provided by RSSI signals [18].

Device-free passive sensing is an emerging technology to sense humans or devices without attaching any additional device to them. The current research field of device-free wireless-based passive sensing is not limited to indoor localization, but also includes human behavior recognition, intrusion detection, which may go far in the future [19]. Generally, the passive indoor localization techniques do not require people to carry specific measuring devices which is more suitable for some special indoor places, such as elderly care, smart homes and security monitoring, etc. [20]. Traditional passive localization systems are mainly based on the coarse-grained RSSI signatures. Received signal strength indicator values usually vary greatly due to multipath even at the same position resulting in a limited localization accuracy [21]. WiFi is now accessible almost everywhere in daily life. Therefore, we can achieve passive indoor localization purposes by applying CSI which can also accomplish high accuracy position without the extra costs required for building infrastructure [22]. 

For instance, Xiao et al. [22,23] proposed the Fine-grained Indoor Fingerprinting System (FIFS) and the Pilot system. FIFS uses the diversity of the original CSI data in the time and frequency domains. It also utilizes a weighted average CSI value based on multiple antennas to improve the accuracy of indoor positioning. Pilot is the first proposal to leverage the temporal stability and frequency diversity characteristics of CSI for developing a CSI-based passive device-free indoor fingerprinting system. The authors of Reference [24] designed and implemented the novel dynamic multiple signal classification (Dynamic-MUSIC) method to detect the subtle reflection signals from the human body to identify the human target’s angle for passive device-free localization. Chaapre et al. [25] designed a new method to generate position fingerprints called CSI-MIMO, which utilizes the phase and amplitude information of all subcarriers. However, the CSI-MIMO method produces only raw CSI data for each subcarrier without any processing and does not take advantage of multiple antennas, which could better reflect the uniqueness of the location. Zhefu Wu et al. [26] designed a naive Bayes classifier-based passive indoor localization system enhanced with confidence level information. Researchers have proposed PhaseFi, a phase fingerprinting system for indoor localization that involved designing a deep network with three hidden layers to train the calibrated phase data, and used weights to represent fingerprints [27]. However, whether all the CSI values collected from the Network Interface Card (NIC) contribute equally to the system accuracy has not yet been thoroughly studied. According to the authors of References [28,29], less significant features could be misleading and confuse the system, and these authors analyzed some factors causing instability in the CSI phase information and proposed linear transformation to remove the interference and extract phase features in order to realize localization. 

So based on the above summary, we can conclude the several technical challenges of passive localization form these studies: (1) How to analyze and process raw CSI data to get stable or robust data features in indoor environments. (2) How to reveal the principle reasons for signal changes due to location difference, and mathematically model the relationship between the CSI fingerprint database and targets. (3) How to automatically cluster the different locations in large-scale fingerprint datasets and in short response times especially with high-accuracy requirements. However, these studies did not fully apply the fine-grained CSI amplitude and phase information, which made it impossible to achieve more accurate positioning. Therefore, it is urgent to solve the problem of passive indoor location with high precision. 

To improve the indoor positioning accuracy and the overall effect, this paper presents FapFi, a passive indoor fingerprint system based on WiFi using the fusion amplitude and phase information of the CSI signal. About the acronym of FapFi, “F” represents the fusion method, “a” represents the amplitude of CSI, “p” represents the phase of CSI, and “Fi” represents the WiFi environment. This method determines the anomalous CSI amplitude values at the subcarrier level and the redundant values at the channel level. The amplitude information is filtered through the above process, and a linear transformation is then applied to extract the calibrated phase information. Finally, the fusion of the feature information is stored in a fingerprint database as a basis for indoor positioning. Compared with the techniques of the above-cited papers, the advantages of the approach described in this paper are as follows:

(1) We proposed to use a fine-grained physic layer information CSI for indoor localization and processed the CSI amplitude and phase data to obtain stable and robust fingerprint features while reducing the signal interference from environmental factors.
(2) We adopted a fusion method to extract the most contributing features from processed CSI data and constructed an efficient fingerprint database.
(3) FapFi applied Naïve Bayesian Classification which satisfies the real-time localization requirement for passive human indoor localization and high-precision positioning in two different environments.
(4) Regarding the performance of localization, we compared FapFi with other methods. We investigated the parameters that affect the performance of positioning accuracy. Experimental results demonstrated that the FapFi system is able to achieve high performance which outperforms a traditional CSI-based system in both environments.

This paper is organized as follows. In Section 2, we briefly introduce the background knowledge of indoor fingerprint localization and CSI. We introduce the amplitude and phase data cleansing methods and focus on the uniqueness and stability of this approach to localization in Section 3.1. Section 3.2 explains the structural design of the positioning system. Section 4 evaluates the selection of the experimental environment and the performance of the system by comparing the experimental results with those of other systems. Conclusions are presented in Section 5.

## 2. Preliminaries

In this section, we will present the background knowledge of indoor fingerprint localization and the FapFi system.

### 2.1. Fingerprint Localization

**Figure 1:**
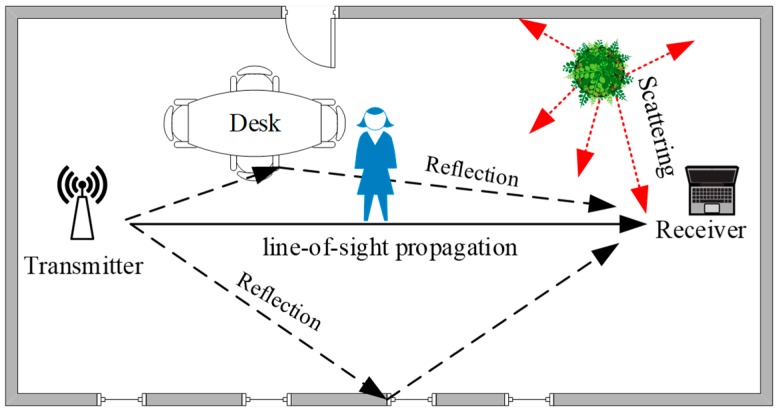
The path of signal transmission in an indoor environment.

As shown in Figure 1, in an indoor environment, the wireless signal is obstructed by obstacles existing in the environment, and the signal is reflected and diffracted to form a multipath effect [30]. Different objects interfere differently with the transmission route, and the personnel are located in different locations; therefore, the signal characteristics are not the same. This difference can be invoked as a fingerprint feature. The process of fingerprint localization involves matching the signal characteristic of an unknown location with the existing information in a fingerprint database to match the best positioning result [31].

The indoor fingerprint localization technology includes two stages: the offline stage and the online stage. The offline stage collects the position data of each reference point in the known area, processes the data to extract a feature, and establishes a relational database of the fingerprint feature of the reference point and the corresponding position coordinates. In the online positioning stage, the data are collected and analyzed in real time; the eigenvalues of the reference points are matched with the fingerprint database by using the matching algorithm, and the exact coordinate position results are thus obtained [32]. 

The traditional localization fingerprint feature is usually identified based on the RSSI signals, the signal-to-noise ratio (SNR), and other such parameters. However, the use of CSI has the advantages of temporal stability, frequency diversity, high stability, and providing a better reflection of the multipath effect in the environment. Furthermore, the applicability of CSI to the field of indoor localization is higher.

### 2.2. Channel State Information

The CSI signal contains more fine-grained physical layer (PHY) information during signal transmission and describes the signal characteristics, such as the amplitude and phase of each subcarrier wave in the channel. The CSI can better describe the communication link properties of the signal from the transmitter to the receiver, which can reflect the existing reflection, diffraction and other interference factors of an indoor environment. The CSI signal represents the combined effects of the channel status, such as scattering, fading, multipath interference, shadowing, and power decay with distance [33]. Currently, we can extract CSI signals in the frequency domain from the channel frequency response (CFR). Each CSI packet includes information such as the timestamp, RSSI, number of antennas, noise, and CSI.

The OFDM system divides the communication channel into several orthogonal subchannels with different frequencies. The received signal after a multipath channel transmission can be expressed as:

(1) $$\vec{Y}=H\vec{X}+\vec{N}$$

where $\vec{Y}$ and $\vec{X}$ denote the signal vectors of the receiver and the transmitter, respectively, and *H* and $\vec{N}$ denote the channel information matrix and additive white Gaussian noise, respectively. The CSI of each subcarrier can be estimated from $\vec{X}$ and $\vec{Y}$ at the receiver, which is expressed as:

(2) $$\hat{H}=\frac{\vec{Y}}{\vec{X}}$$

where $\hat{H}$ represents the CFR of each sub-channel. The CSI can be divided into different subcarrier groups according to the different hardware drivers at the receiver, and the CSI matrix can be expressed as:

(3) $$H=\left[H_{1},H_{2},\cdots ,H_{N}\right]$$

where *N* is the number of subcarrier divisions based on the hardware drivers of wireless network card. For example, *N* = 56 for a 20-MHz bandwidth channel, and *N* = 114 for a 40-MHz bandwidth channel. We used a 20-MHz bandwidth channel in this paper, thus the index of the subcarriers was 56. The CSI for each subcarrier is expressed as:

(4) $$H_{i}=\left|H_{i}\right|e^{j\sin(∠H_{i})}$$

where $\left|H_{i}\right|$ and $∠H_{i}$ are the amplitude and phase of the *i*-th subcarrier, respectively.

The CSI extracted from the experimental platform is an m×n×k complex matrix, where *m* and *n* denote the numbers of antennas at the transmitter and the receiver, respectively, and *k* denotes the number of subcarriers.

**Figure 2:**
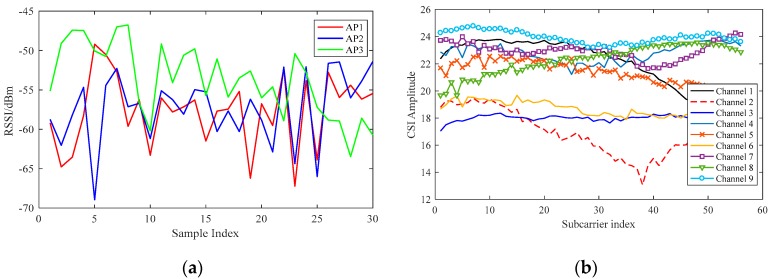
Comparison between (**a**) received signal strength indicator (RSSI) and (**b**) channel state information (CSI) signals.

The comparison between the CSI and RSSI signals is shown in Figure 2. In order to compare the effects of using CSI and RSSI data as data features, we randomly selected a test point in our environment and collected a set of CSI data packets from the Atheros 9380 network card. Meanwhile, we used Android mobile applications to collect RSSI data in the same environment in contrast to CSI data. The purpose of using mobile applications is to collect real-time RSSI data. The amplitude information of the CSI from three transmitter antennas and three receiver antennas can be analyzed using CSI data packets. It can be observed in the figure that the CSI signal changes more smoothly and steadily in the same environment and that the RSSI signal is prone to fluctuations. This comparison also verifies that the CSI signal has better spatial discrimination and stability over time, which is more suitable as a fingerprint for indoor localization.

### 2.3. Naive Bayesian Classification

The naive Bayes method is a classification algorithm based on Bayes’ rule. The algorithm is easy to implement, and the overall complexity and usage of time and space is low [34]. In practical applications, this method has the advantages of dealing with multiple types of problems and making faster matches [35]. The principle is shown in Equation (5):

(5) $$P(A|B)=\frac{P(B|A)\times P(A)}{P(B)}$$

where *P* is the probability and P(A|B) is the conditional probability; *A* can be understood as a category, and *B* as a feature. The naive Bayesian classification is based on two basic assumptions: (1) the features are independent from each other; (2) each feature has the same probability distribution 26 [34].

The naive Bayesian classifier model is described as follows. Suppose the training set contains *m* classes $C=\{C_{1},C_{2},\cdots ,C_{m}\}$ and *n* conditional attributes $X=\{X_{1},X_{2},\cdots ,X_{n}\}$. Assuming that all of the conditional attributes *X* are children of a class variable *C*, if $P(C_{i}|X)>P(C_{j}|X)$, and only if (1≤i,j≤m,i≠j) holds, then assign a given sample to be classified $X=\{x_{1},x_{2},\cdots ,x_{n}\}$ to class $C_{i}(1\leq i\leq m)$. According to Equation (5), the posterior probability of class $C_{i}$ is:

(6) $$P(C_{i}|X)=\frac{P(C_{i})P(X|C_{i})}{P(X)}$$

In the classification problem of this paper, *C* indicates the set of location points to be classified and *X* represents the set of CSI fingerprint feature data at a location point. The specific application will be discussed in Section 3.2.

## 3. FapFi System Design

**Figure 3:**
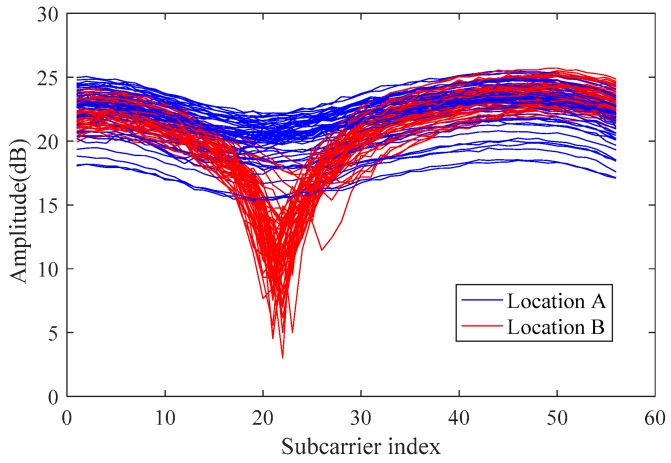
CSI amplitude.

The CSI-based fingerprinting mainly needs to meet two requirements: (1) the signal should be fixed as stable as possible when the CSI signal is at a certain point; and (2) the CSI signals collected at different points should be easily distinguished to distinguish different locations. As shown in Figure 3, the red and blue CSI signal amplitudes were collected at different points and can reflect the stability and distinguishability of the CSI. Furthermore, the figure also reflects that due to several factors such as strong multipath effect, the position of the furniture and so on in the environmental interference, there is considerable noise in the original CSI signal. The FapFi method fuses processed CSI amplitude and phase data to obtain stable and robust fingerprint features. The most important thing is FapFi satisfied timeliness, accuracy, and stability requirement of passive localization. The following content will introduce our main idea of designing FapFi method.

To reduce the CSI signal interference caused by the indoor environment, we need to solve two key factors including data preprocessing and feature extraction, thereby we try to ensure that the experimenter is absolutely static and the test environment is stable when collecting data in the offline and online stage, which can reduce other interference factors such as changes in furniture position. Secondly, we choose a relatively empty environment to collect a large number of experimental data for analysis and then we utilize the phase characteristics of the CSI to make up for the shortcomings of the amplitude. In this paper, we separately processed the amplitude and phase information, and the processed features were then fused to improve the positioning accuracy.

### 3.1. Data Sanitization 

In this section, we describe the data cleansing step and utilize the fusion feature to build a unique, robust fingerprint.

#### 3.1.1. Amplitude Sanitization

During the process of indoor localization, the tester was required to stand still, and changes in the signal due to slight activity of the human body will thus be concentrated at frequencies lower than any abnormal frequencies [36]. Based on this analysis, the filter of the amplitude can effectively remove the irrelevant signal frequencies caused by non-human activities, thereby eliminating the noise interference caused by the amplitude of the subcarriers while preserving the effective amplitude data.

**Figure 4:**
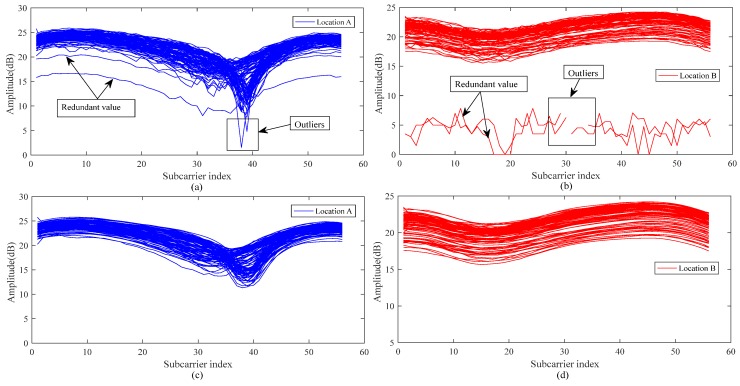
(**a**,**c**) unprocessed CSI amplitudes; (**b**,**d**) processed CSI amplitudes.

We had a researcher remain standing at the test point and continuously collected 100 CSI packets, taking the amplitude of one of the links. The results are presented in Figure 4a,b. The redundant values and outliers of the CSI signals are indicated by the arrows in the figure. To judge the abnormal values present in the collected CSI signal amplitudes, we chose to calculate the standard deviation for the collected data packet at the subcarrier level. The standard deviation describes the degree to which the amplitude deviates from the mean value during the data acquisition stage, so it can be used to judge the outliers of the amplitude. Regarding the redundant values, we considered the relationship of the outlier data packets in the filtering process to ensure the correlation between the data in the channel level, which means that during the entire communication process we processed the redundant values. The specific steps in filtering the CSI amplitude characteristics are shown below.

Step 1: Calculate the mean value $\bar{Am^{i}}$ of the *i*-th subcarrier of *k-*th data packet according to Equation (7):

(7) $$\bar{Am^{i}}=\frac{1}{N}\sum_{k=1}^{N}Am_{k}^{i}$$

where *N* is the number of samples, and i∈[1,56] is the subcarrier index.

Step 2: Calculate the standard deviation of the *i*-th subcarrier from Equation (8):

(8) $$\sigma_{i}=\sqrt{\frac{1}{N}\sum_{k=1}^{N}{\left(Am_{k}^{i}-\bar{Am^{i}}\right)}^{2}}$$

*i* is the index of the subcarriers, so we can get the $V=\left[\sigma_{1},\sigma_{2},\cdots ,\sigma_{55},\sigma_{56}\right]$ which is a variance matrix of the 56 subcarriers. 

Step 3: Assuming that the data packet to be filtered is k, the CSI amplitude values are ${\left|Am\right|}_{k-1}^{i}$ and ${\left|Am\right|}_{k+1}^{i}$ for each adjacent data packet k−1 and k+1, respectively. According to Equation (8), the filtered amplitude ${\left|Am\right|}_{filter}^{i}$ is calculated by averaging the three amplitude data values.

(9) $${\left|Am\right|}_{filter}^{i}=\frac{1}{3}({\left|Am\right|}_{k-1}^{i}+{\left|Am\right|}_{k}^{i}+{\left|Am\right|}_{k+1}^{i})$$

Step 4: For the processed amplitude $Am_{filter}$, the covariance matrix $Cov(Am_{filter},Am)$ of $Am_{filter}$ and $\bar{Am}$ is calculated. If the variation trends of the two variables are consistent, the covariance is positive. If the two variables change in opposite directions, the covariance between the two variables is negative, which is considered redundant and removed from the packet.

As shown in Figure 4a,b, after the above steps determination, we can observe that the redundant values are usually deviated from normal values which means that the covariance between redundant values and normal values are negative. Outliers are usually caused by problems such as data packet loss or data peak anomaly.

After processing the CSI amplitude values shown in Figure 4c,d below, the filtered CSI amplitude values are smoother, with the redundancy caused by various factors effectively removed and the abnormal values caused by environmental factors filtered out. In the course of the experiment, we adopted a large number of CSI packets, so the problem that outlier filtering processing over-sensitive and inaccurate data can be avoided.

#### 3.1.2. Phase Sanitization

The phase information is seldom used for CSI-based indoor localization schemes mainly because the hardware is not good enough to measure the true phase [37]. Therefore, researchers generally use the amplitude value of the CSI to locate an object, and the phase is rarely used in indoor localization. The phase measurement $∠\overset{\wedge }{Ph_{i}}$ of the *i*-th subcarrier can be expressed by Equation (10):

(10) $$∠\overset{\wedge }{Ph_{i}}=∠Ph_{i}+2\pi \frac{k_{i}}{N}t+\beta +Z$$

where $∠Ph_{i}$ is the true phase value, *t* is the timing offset between the transmitter and receiver, β is the phase offset caused by the carrier frequency offset, and Z is the measurement noise, while $k_{i}$ stands for the *i*-th subcarrier index. In the Atheros platform k∈(1,56), N is the number of fast Fourier transform (FFT) samples, and N is 64 in the IEEE 802.11 a/g/n protocol. Due to the above factors t, β, and Z, ordinary WiFi NICs are unable to obtain the true phase values.

To mitigate the impact of random noises and extract the available phase information, we measure changes of CSI signal phase in an indoor environment and perform a linear transformation on the raw phase data, as recommended in References [28,36,38,39] et al. The main idea is to apply a linear transformation to the original phase value and eliminate the interference factors t and β by considering the phase across the entire frequency band.

First, the two formulas for slope *a* and intercept *b* are defined as follows:

(11) $$a=\frac{∠\overset{\wedge }{Ph_{i}}-∠\overset{\wedge }{Ph_{1}}}{k_{i}-k_{1}}=\frac{∠Ph_{i}-∠Ph_{1}}{k_{i}-k_{1}}-\frac{2\pi }{N}\Delta t$$

(12) $$b=\frac{1}{n}\sum_{j=1}^{n}∠Ph_{j}-\frac{2\pi \Delta t}{nN}\sum_{j=1}^{n}k_{j}+\beta$$

Equations (11) and (12) are based on the assumption of the linear transformation method. In Equation (11), Δt is the corresponding difference of timing offset. According to the IEEE 802.11n specification, the subcarrier frequency is symmetric, which indicates $\sum_{j=1}^{n}k_{j}=0$, so the error term Δt can be further removed and *b* can be expressed as $b=\frac{1}{n}\sum_{j=1}^{n}∠Ph_{j}+\beta$, which neglects the influence of the measurement noise Z. β is the equivalent timing offset of the receiver caused by the device, which in fact cannot be eliminated but can only reduce its impact. By subtracting the linear term $ak_{i}+b$ from the original phase $∠\overset{\wedge }{Ph_{i}}$, part of the random phase shifts can be removed. We can then obtain the transformed phase denoted by $∠\tilde{Ph_{i}}$:

(13) $$∠\tilde{Ph_{i}}=∠\overset{\wedge }{Ph_{i}}-ak_{i}-b=∠Ph_{i}-\frac{∠Ph_{n}-∠Ph_{1}}{k_{n}-k_{1}}k_{i}-\frac{1}{n}\sum_{j=1}^{n}∠Ph_{i}$$

**Figure 5:**
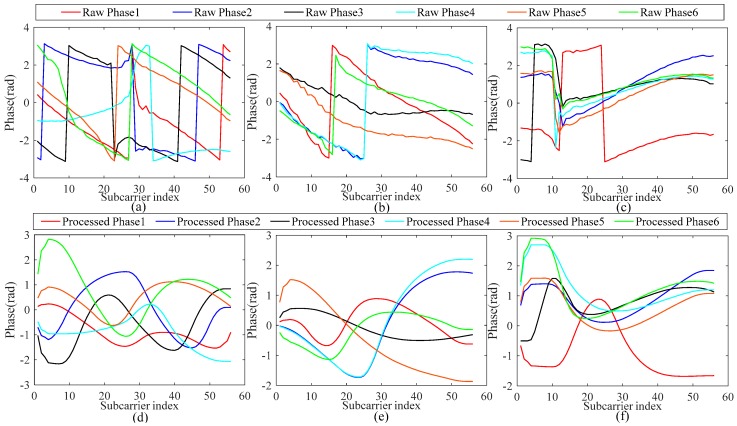
(**a**–**c**) Original phase for the 6 data packets in channels 1, 5, and 9; (**d**–**f**) corresponding phase processing result for a set data packets in different channels.

Figure 5a–c depicts the raw phase features of channel 1, channel 5, and channel 9, respectively, with six data packets selected for each channel to illustrate the effect that CSI phase information is unstable in the environment. The CSI phase was also affected by the sampling frequency offset and the carrier frequency offset; while there has been some research conducted on this, we did not discuss these two factors here, we only calibrated the phase information through the handy linear transform method to apply phase features in the fingerprint database. After linear processing, the instability of the phase was effectively reduced and the phase features could be converted into ordered and analyzable data. Figure 5d–f shows the processed phase of the six CSI data packets; the raw phase had a large fluctuation range and the processed phase had a smoother, small range especially where the original phase fluctuated. This indicates that the proposed linear transformation can remove the phase offset. Overall, the calibrated phase is stable enough for indoor localization requirements and solved the problem of data instability, which is the basis for extracting the high-robustness and high-efficiency fingerprint feature values by fusion of the amplitude and phase features.

### 3.2. System Architecture 

**Figure 6:**
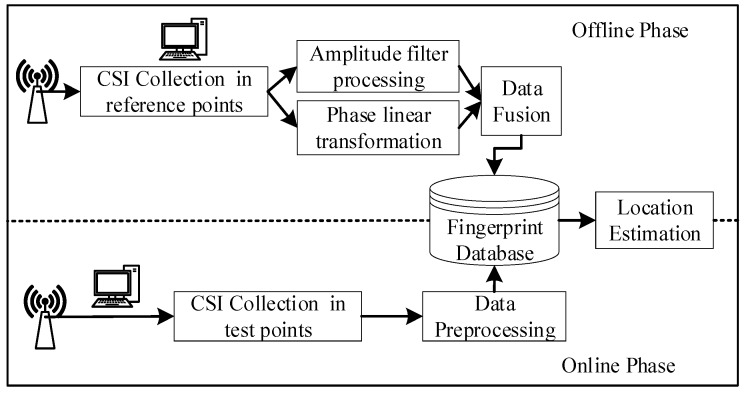
Positioning system architecture.

The FapFi system architecture is shown in Figure 6. In the offline phase, P reference points are selected in the target area L; the position information of each reference point is known, and the CSI values of all the reference points at times Q are collected to form the original position fingerprint F:$F=\left|\begin{array}{cccc} {\mathrm{csi}}_{11} & {\mathrm{csi}}_{12} & \cdots & {\mathrm{csi}}_{1Q}\\ {\mathrm{csi}}_{21} & {\mathrm{csi}}_{22} & \ldots & {\mathrm{csi}}_{2Q}\\ \vdots & \vdots & \vdots & \vdots \\ {\mathrm{csi}}_{P1} & {\mathrm{csi}}_{P2} & \ldots & {\mathrm{csi}}_{PQ} \end{array}\right|$. Equation (4) shows that $csi_{PQ}=H_{PQ}$; that is, the amplitude and phase information of each point are obtained.

Assuming that the processed amplitude is ${\left|Am\right|}^{new}$ and the phase is $∠\tilde{Ph}$, a linear weighted fusion is performed on ${\left|Am\right|}^{new}$ and $∠\tilde{Ph}$ to obtain a new feature $csi^{\prime }$:

(14) $$csi^{\prime }=w_{1}{\left|Am\right|}^{new}+w_{2}∠\tilde{Ph}$$

Equation (14) can be used to calculate the percentage of the amplitude or phase in the new fingerprint database, and it represents the proportion of the amplitude and phase information of a fingerprint feature taken at a test point after it has been processed. It is convenient and simple to calculate using the linear weighted fusion method. Where $w_{1}$ and $w_{2}$ are the feature fusion weights, which satisfy the constraints of Equation (15), and $csi^{\prime }$ is the fused feature.

(15) $$\{\begin{array}{c} w_{1}+w_{2}=1\\ \begin{array}{l} 0<w_{1}<1\\ 0<w_{2}<1 \end{array} \end{array}$$

The fingerprint feature of a test point is only composed of CSI amplitude and phase, and its condition is constrained by Equation (15). In advance, set $w_{1}=w_{2}=0.5$ and adjust the weight of the amplitude and phase in the fingerprint database according to the changes in the environment and the positioning accuracy; that is, dynamically adjust the $w_{1}$ and $w_{2}$ assignments, the details will be discussed in Section 4.2.3. 

The original amplitude and phase information of P reference points are subjected in *Q* times to the above processing steps to form a new signal feature $cs{i_{PQ}}^{\prime }$, thereby developing the fingerprint database $F^{\prime }$ in the offline phase and forming the mapping relationship between the points in the spaces L and F. At present, there is a ubiquitous limitation of fingerprint positioning. The fingerprint database is only available for the environment and targets we have built. If the environment and target are changed, the database needs to rebuild.

In the online phase, the signal features of an unknown position $l_{i}\left(x_{i},y_{i}\right)$ are collected, and the amplitude and phase information of the point is obtained after the data processing. The naive Bayesian classification algorithm is matched with the fingerprint database $F^{\prime }$ online to output the best result that estimates the location of the test point $l_{i}$.

For prior probabilities of random locations $l_{i}\in L$ in a space to be the same and known, $P(l_{i}|x)$ is equivalent to calculating the maximum posterior probability of $P(x|l_{i})$, where x denotes the term to be classified in naive Bayesian algorithm:

(16) $$P(l_{i}|x)=\frac{P(l_{i})P(x|l_{i})}{P(x)}$$

Assuming that *P(l_i_)* and *P(x)* are known, the probability estimation obeys the Gaussian distribution, $P(x|l_{i})\sim N(\delta ,\theta )$, δ and θ are the mean deviation mean(i) and mean square error std(i), respectively. The maximum probability value $P(x|l_{i})$ of location $l_{i}$ is solved by Equation (17), and the category with the largest posterior probability is taken as the matching result of the unknown point $l_{i}$.

(17) $$l_{i}\leftarrow \arg\max P\left(l_{i}|x\right)=\arg\max\frac{P(L_{i})P(x|L_{i})}{P(x)}$$

The predictive ability of the Bayesian classification algorithm is related to the completeness of the training samples. After adopting the correlation processing method of this algorithm, the training samples are more representative, which can lead to more accurate location results.

## 4. Experiment Validation

In this section, we show the detailed results obtained in the experimental environment and the data collection method of our proposed system. Afterward, we will evaluate the performance of our proposed system in various scenarios and compare the resulting location errors in different environments with several benchmark schemes.

### 4.1. Experimental Setup 

**Figure 7:**
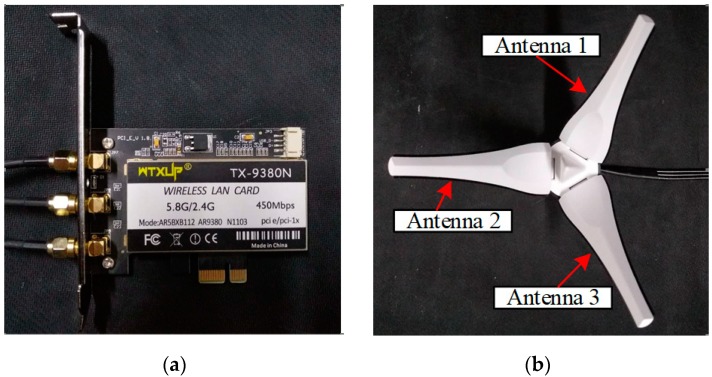
Experimental equipment (**a**) Atheros 9380 network cards; (**b**) NIC antennas

In FapFi system, two desktop computers with Atheros 9380 network cards were employed in the experimental environment. The Ubuntu10.04LTS operating system (Canonical, London, England), which is driven by a custom kernel and modified wireless network card firmware, was installed in two desktop computers. One PC was equipped with an Intel Core i3-4150 CPU (Intel, Santa Clara, CA, USA) that functioned as a transmitter, and the other PC worked as a receiver. The Atheros NIC programs used the Atheros-CSI-Tool, which is an open source driver developed by Xie et al. [40]. After the above procedures, the Atheros 9380 wireless network card can extract the CSI signals between the receiver and the transmitter. As shown in Figure 7, the Atheros 9380 NIC we used had three antennas in the experiment. 

**Figure 8:**
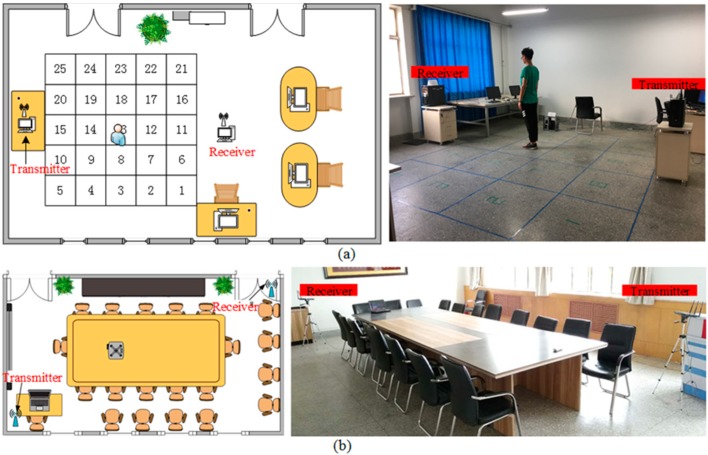
Experimental scenarios: (**a**) floor plan in laboratory; (**b**) floor plan in meeting room and experimental equipment.

Our techniques were tested in two different indoor sceneries. The first testing scenery was a relatively empty laboratory which has been commonly used in many previous studies. As shown in Figure 8a, there were enormous line of sight (LOS) receptions which suffer less from multipath effects to validate the effectiveness of the system. In the 9 m × 6 m area, 25 deployment square areas were deployed. Each square area was 0.8 m × 0.8 m, the center of the square was the corresponding position coordinate of the reference point in the process of building the fingerprint database, and the receiver antennas was 4.5 m apart from the transmitter antennas and the antenna height was 1.2 m. 

The approaches proposed in previous studies were primarily tested in the first environment which contained less noise and interference. The second testing scenery was a meeting room with metal tables, chairs, and desktop computers. Hence, this environment had a relatively large number of multipaths in more extreme environments to further validate our method with previous work. Figure 8b shows the environment of the second testing location, which was quite crowded, and most of the LOS paths were blocked. The experimental equipment configuration was as described above. We arbitrarily chose test points within the 8 m × 6 m area. The receiver antennas were 6.5 m apart from the transmitter antennas, and the antenna height was 1.2 m. In both testing cases, the direction and position of the experimenter’s stance remained unchanged during the data collection and estimation.

### 4.2. Experimental Analysis

In this section, we discuss various parameters and evaluate their impact on the performance of our system, such as the selected fingerprint features, the number of antennas deployed, and test samples. We illustrate the results in the rest of paper.

The localization effect of the algorithm can be measured by two indexes: location accuracy and average error distance.

Location Accuracy: The ratio of the correct location prediction category to the total number of tests.

Average Error Distance: Assuming that the number of tests is *N*, the *i*-th estimated position coordinates $\hat{L}({\hat{x}}_{i},{\hat{y}}_{i})$, the actual position coordinates $L(x_{i},y_{i})$, the error distance can be obtained by Euclidean distance between $\hat{L}$ and L, and the average error distance $D_{error}$ can be expressed by Equation (18):

(18) $$D_{error}=\frac{1}{N}\sum_{i=1}^{N}\sqrt{{\left({\hat{x}}_{i}-x_{i}\right)}^{2}+{\left({\hat{y}}_{i}-y_{i}\right)}^{2}}$$

#### 4.2.1. Impact of Selected Fingerprint Features

**Figure 9:**
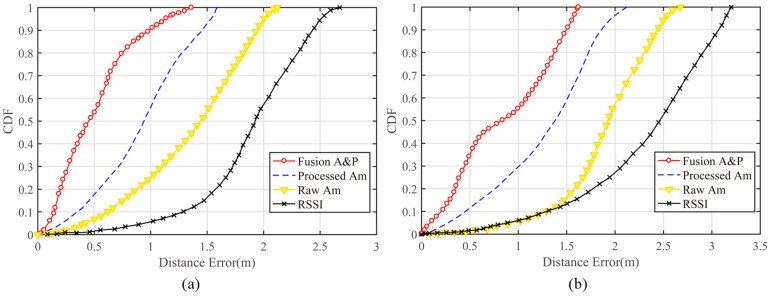
**Cumulative Distribution Function** (CDF) of localization error of different fingerprint features in different environment (**a**) in the laboratory and (**b**) in the meeting room.

In the experiment, the tester (with a height of 1.83 m) stood in the test area to collect the test data for different test points. We separately analyzed the results obtained using the amplitude and phase fusion, the processed amplitude, the raw amplitude, and RSSI fingerprinting. Because the original CSI phase data were not useful for the location, the original phase data were not analyzed in this experiment. The location error for the cumulative distribution function of the fingerprint database constructed with the various feature data is shown in Figure 9. As seen from Figure 9a, using CSI signals as the fingerprint feature for indoor localization is better than using RSSI signals in the laboratory environment. A positioning accuracy of 1.8–2.5 m could be achieved with RSSI signals, and using the processed amplitude as the eigenvalue resulted in sub-meter-level positioning accuracy. Through the fusion of the processed amplitude and phase data, the positioning error for 90% of the test points could be reduced to within 1 m, with a probability that 54.6% of the test points were within 0.5 m. The overall positioning accuracy was affected by the complex environment in the meeting room; the tables and other objects obstructed most LOS paths and magnified the multipath effect. In the more complex environment, a positioning accuracy of 2–3 m could be achieved with RSSI signals, and using the amplitude and phase fusion method resulted in a 1-m distance error for over 56% of the test points. The overall performance of this fusion method was better than any other method, which greatly enhanced the CSI-based indoor localization accuracy and verified the validity of the amplitude and phase information processing proposed in this paper. Figure 9 also illustrates that the unprocessed CSI values will affect the accuracy of the decline, and the amplitude and phase data cleansing methods proposed in this paper are effective for improving positioning performance.

#### 4.2.2. Impact of Antennas and the Number of Packets

With the indoor location algorithm based on WiFi, the numbers of deployed antennas, training samples, and testing samples are key factors affecting the positioning accuracy when using the fingerprint location method. The numbers of transmitting and receiving antennas determine the number of channels, and the optimal combination of antenna numbers can achieve highly precise positioning accuracy. To address this issue, in this experiment, we deployed the receiver and transmitter shown in Figure 7, and we set m=3,n=1, m=3,n=2, and m=3,n=3, where *m* and *n* denote the numbers of antennas at the transmitter and the receiver, respectively. So, we can get a combination of 3, 6, and 9 channels, and it is worth noting that the concept of channels are communication links which act as signal transmission paths between transmitters and receivers. Then we evaluated six combinations: 1000 training data packets/200 testing data packets, 500 training data packets/200 testing data packets, 500 training data packets/100 testing data packets, 250 training data packets/100 testing data packets, 100 training data packets/100 testing data packets, and 100 training data packets/50 testing data packets, each performed with 3, 6, and 9 channels. A total of six test samples were experimentally tested to analyze the corresponding average positioning error in a laboratory scenario.

**Figure 10:**
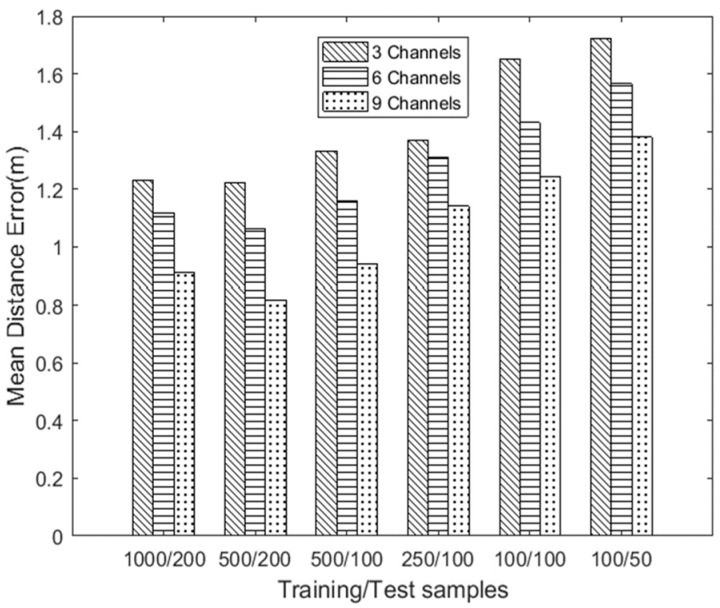
Positioning accuracy of different antennas and packet numbers.

Figure 10 shows that, without considering the impact of data packets, the average distance error of positioning was the highest among all the sample combinations when only three channels were used. As the number of antennas increased, the positioning accuracy gradually increased.

Next, we analyzed the influence of the number of training samples and the number of test samples on the positioning accuracy. The average positioning error for the 1000/200 data and the 500/200 data combinations was relatively small. However, for the 1000/200 combination, the main reason for the slight performance degradation compared to the 500/200 combination was that the 1000/200 combination required a longer time to train the classification model; in addition, there was overfitting when the training dataset needed to extract and analyze more amplitude and phase information in data processing. For the 250/100, 100/100, and 100/50 data sets, the localization effect was not ideal when the data samples were relatively few. Based on the experimental results, 500 training packets/200 test packets with nine channels were used for subsequent experiments.

#### 4.2.3. Impact of Feature Fusion Weights

In Section 3, we used a linear weighted fusion in constructing the fingerprint database, with the feature fusion weights $w_{1}$ and $w_{2}$ representing the proportion of the amplitude and phase information, respectively, in the fingerprint database. After data fusion, the feature data not only effectively reduced the data dimensions, but also makes full use of CSI fine-grained data, and can more reasonably allocate the proportion of amplitude and phase features in the fingerprint database. 

In order to eliminate redundant information and generate more distinctive fingerprint features, we dynamically adjusted the weights according to the constraints of weights. In the initial stage, we set $w_{1}=w_{2}=0.5$ and tested it 10 times for debugging. We can calculate the maximum positioning error $\max D_{error}$ and the minimum positioning error $\min D_{error}$ by Equation (18), and then calibrate next weights according to the latest positioning error. The weight value of amplitude $w_{1}$ is calculated by Equation (19) and the weight value of phase *w*_2_ was obtained. In order to ensure the accuracy of positioning, we specified that the values of *w*_1_ and *w*_2_ must not be less than 0.1 or greater than 0.9. Also, if $D_{error}>\max D_{error}$ or $D_{error}<\min D_{error}$, the value of $\max D_{error}$ or $\min D_{error}$ was updated immediately.

(19) $$w_{1}=\frac{D_{error}-\min D_{error}}{\max D_{error}-\min D_{error}}$$

**Figure 11:**
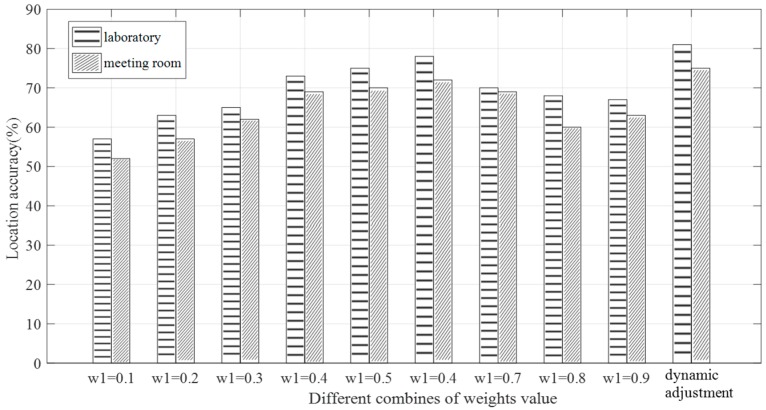
Influence of feature fusion weights on positioning accuracy in different environments.

To analyze the influence of the feature fusion weight values $w_{1}$ and $w_{2}$ on the localization result, we used nine groups of control data to carry out the experiments, the case of $w_{1}=1$ or $w_{2}=1$ was already discussed in Section 4.2.1. We ran 100 tests in the laboratory environment and the meeting room environment to calculate the location accuracy under different conditions. The results are shown in Figure 11. When using the dynamic adjustment scheme, the accuracy of positioning in laboratory and meeting room environments reached 81% and 75%, respectively. It can be observed that the positioning accuracy of the dynamic adjustment weight method in different environments was higher than that setting the fixed weight. Although the dynamic adjustment weight value method was not obvious for the improvement of the positioning accuracy, it provided flexibility for the improvement of the positioning error. The results of these experiments indicate that the phase information can compensate for shortcomings when the amplitude information performs poorly at the more accurate positioning distance. The appropriate adjustment of the weights of the amplitude and phase in the fingerprint database can reasonably utilize the fine-grained data features of CSI and improve the positioning accuracy.

#### 4.2.4. Impact of the Number of Reference Points

In the fingerprint localization algorithm, the number of reference points used in the fingerprint space in the offline stage is also an important parameter that affects the positioning performance. More reference points result in a better positioning effect. In this paper, to analyze the impact of the number of reference points on the localization algorithm, according to the planar design in Figure 8a, we selected 25, 50, 75, and 100 reference points as the parameter variables to analyze the effect on the results and the execution time of the algorithm in a laboratory scenario.

**Figure 12:**
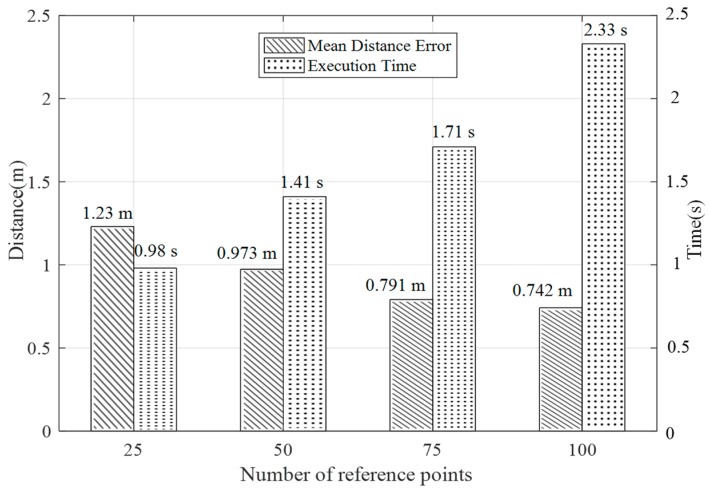
Influence of reference points on positioning accuracy.

As shown in Figure 12, in the selected experimental region, when the number of reference points was selected in the range of 75 to 100, the positioning accuracy was approximately 0.75 m, and the execution time of the algorithm was approximately 2 s. An analysis based on these two aspects yields an optimal positioning result. In terms of the overall analysis, the number of reference points was positively correlated with the positioning accuracy. As the number of reference points increases, the positioning error gradually decreases, while the time taken for offline fingerprint database acquisition was not considered. Compared with the online phase algorithm, increasing the number of reference points will increase the algorithm execution time.

#### 4.2.5. Overall Performance

To compare the effects of different localization algorithms, we compared our proposed localization method with the PhaseFi, CSI-MIMO, and FIFS systems. We collected 500 training packets/200 test packets from test locations with nine channels and randomly selected test locations in the environments. We validated our technique under two environmental conditions.

**Figure 13:**
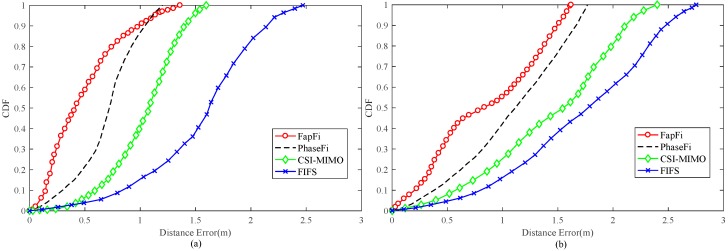
CDF of distance errors using different localization methods in different environments: (**a**) in the laboratory and (**b**) in the meeting room.

As shown in Figure 13a, in the proposed system, 58.8% of the test position errors were controlled within 0.5 m, and 91.1% of the errors were below 1 m, while PhaseFi ensured that approximately 21% of the test positions had errors below 0.5 m and 87% were below 1 m. The overall performance of the CSI-MIMO and FIFS systems in the test environment was not good, mainly because FIFS only uses the diversity of the original data in the time domain and the frequency domain as the fingerprint database and because CSI-MIMO uses the multi-antenna mode to collect the CSI data. Neither of these systems further processed the data, thus leading to an overall positioning accuracy of greater than 1 m. For PhaseFi, which uses neural network algorithms, the advantages were reflected by positioning accuracies in the range of 0.7–1 m. FapFi had a higher positioning efficiency than PhaseFi within 0.3–0.7 m because FapFi adopts the fusion method to utilize both the amplitude and phase features of the CSI.

The meeting room environment contrasts with the laboratory environment. As shown in Figure 13b, although multipath propagation degrades the accuracy of the localization, our proposed method is robust enough to maintain accuracy in the meeting room. The positioning accuracy of FapFi within 1 m was still very good, whereas with PhaseFi, only 42% of the test points had an estimation error under 1 m, while for CSI-MIMO and FIFS, the values were 27.5% and 19%, respectively. Our proposed system performed better than the other algorithms within the range of 1–1.5 m. It can be concluded from the experiment that the use of phase features can compensate for the shortcomings of using only the amplitude feature for the fingerprint database in the traditional method and can achieve a better positioning effect. The fundamental reason for this is because of the way in which we processed the data, which was very effective.

**Figure 14:**
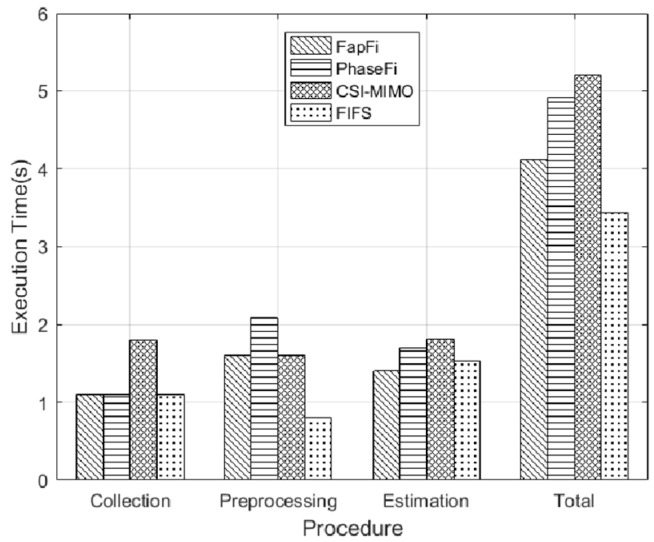
Execution time of different localization schemes.

We also analyzed the average execution time of different systems for a further comparison. The execution times of different systems included reference point CSI data acquisition time, data processing time, and position estimation time. Reference point CSI data acquisition time is offline stage data acquisition time, and data processing time is calculated by the time-consuming in the data sanitization stage and position estimation time is the response time of the different systems in the online stage. Figure 14 shows the execution time required by various algorithms for a single test point. In the CSI collection procedure, FapFi, PhaseFi, and FIFS need the same amount of time to collect the data. The CSI-MIMO uses multiple antennas for the training dataset, and thus requires more collection time. In the processing stage, PhaseFi takes 2.1 s longer than the other algorithms, and only the FIFS method takes less time to build the fingerprint database because FIFS uses a weighted average value of different antennas and the coherence bandwidth to reduce the complexity of the algorithm. During the online phase, the estimation procedure of the proposed system is 1.41 s faster than the other systems. The main reason for this is that we used the naive Bayesian algorithm, and the advantage of the naive Bayesian algorithm is that it enables a viable and effective classification of large data sets in a relatively short time. The overall results show that the total execution time of FapFi is 4.11 s and that of PhaseFi, CSI-MIMO, and FIFS is 4.91 s, 5.21 s, and 3.43 s, respectively. Due to the superiority of the FIFS system in the preprocessing stage, FIFS takes less execution time, but the FIFS system positioning accuracy is not high. In contrast, FapFi can achieve greater positioning accuracy in a shorter amount of time than the other algorithms in terms of location accuracy and execution time.

## 5. Conclusions

We considered the multipath effect and time-variance of CSI signals in indoor environments. In this paper, we proposed FapFi, a passive indoor localization system leveraging the CSI amplitude and phase features to yield the fingerprint. FapFi takes advantage of the physical layer of the CSI in the widely used, off-the-shelf WiFi infrastructure and aims to achieve a high-precision positioning effect. To eliminate the interference caused by signals in the indoor environment, we filtered the CSI amplitude information and linearly converted the phase information. Furthermore, the performance of our system can be further enhanced by utilizing the CSI amplitude and phase information after fusion processing. We validated the performance of our system in both a laboratory and a meeting room. According to the experimental results, the average positioning error was approximately 0.5 m in the laboratory and within 1.2 m in the meeting room. Then, compared with the CSI-based localization method, our presented fingerprint had higher accuracy, which verifies that the proposed fusion of the CSI amplitude and phase data can effectively improve the accuracy of indoor positioning. 

In future work, the flexibility of the system is expected to improve step by step, and the timeliness of the fingerprint database will be further improved. More importantly, the experimental deployment and testing are very complex and time-consuming as of now. With the release of the Atheros CSI Tool OpenWRT version, our future research work will find a better way to solve the site survey or fingerprint databases construction, such as a crowdsourcing approach or other machine learning algorithms [41,42,43,44].
